# Methanol Generates Numerous Artifacts during Sample Extraction and Storage of Extracts in Metabolomics Research

**DOI:** 10.3390/metabo8010001

**Published:** 2017-12-22

**Authors:** Claudia Sauerschnig, Maria Doppler, Christoph Bueschl, Rainer Schuhmacher

**Affiliations:** Center for Analytical Chemistry, Department of Agrobiotechnology (IFA-Tulln), University of Natural Resources and Life Sciences, Vienna (BOKU), Konrad-Lorenz-Straße 20, 3430 Tulln, Austria; claudia.sauerschnig@boku.ac.at (C.S.); maria.doppler@boku.ac.at (M.D.); christoph.bueschl@boku.ac.at (C.B.)

**Keywords:** untargeted metabolomics, stable isotopic labeling (SIL), acidification, sample storage, plant metabolomics

## Abstract

Many metabolomics studies use mixtures of (acidified) methanol and water for sample extraction. In the present study, we investigated if the extraction with methanol can result in artifacts. To this end, wheat leaves were extracted with mixtures of native and deuterium-labeled methanol and water, with or without 0.1% formic acid. Subsequently, the extracts were analyzed immediately or after storage at 10 °C, −20 °C or −80 °C with an HPLC-HESI-QExactive HF-Orbitrap instrument. Our results showed that 88 (8%) of the >1100 detected compounds were derived from the reaction with methanol and either formed during sample extraction or short-term storage. Artifacts were found for various substance classes such as flavonoids, carotenoids, tetrapyrrols, fatty acids and other carboxylic acids that are typically investigated in metabolomics studies. 58 of 88 artifacts were common between the two tested extraction variants. Remarkably, 34 of 73 (acidified extraction solvent) and 33 of 73 (non-acidified extraction solvent) artifacts were formed *de novo* as none of these meth(ox)ylated metabolites were found after extraction of native leaf samples with CD_3_OH/H_2_O. Moreover, sample extracts stored at 10 °C for several days, as can typically be the case during longer measurement sequences, led to an increase in both the number and abundance of methylated artifacts. In contrast, frozen sample extracts were relatively stable during a storage period of one week. Our study shows that caution has to be exercised if methanol is used as the extraction solvent as the detected metabolites might be artifacts rather than natural constituents of the biological system. In addition, we recommend storing sample extracts in deep freezers immediately after extraction until measurement.

## 1. Introduction

Typical metabolomics studies comprise of several steps such as cultivation, harvest, quenching of sample material (e.g., with liquid nitrogen or with cooled organic solvents), metabolite extraction, GC/LC-MS or NMR analysis, data processing and statistical data evaluation [1]. Many of these steps may produce artifact compounds, which have been defined by De Haven et al. [2] as “any chemical whose presence can be attributed to sample handling and processing and not originating from the biological sample.” For example, during most sample preparation steps releasing agents and softeners from plastic ware (e.g., tubes) can be washed out and consequently detected as artifacts. Another step which can lead to artifacts is incomplete quenching. Quenching is meant to stop enzymatic activity and this step is mandatory to preserve the state of the metabolome after sampling. Especially primary metabolites, which can have a rapid turnover (e.g., less than 1 s) [3] (p. 40), may change rather fast when the quenching step is incomplete or too slow. Hence, derivatives formed after sampling by incomplete quenching could be erroneously mistaken as true biochemical constituents and thus may result in wrong biological conclusion of the metabolomics experiment.

Another sample preparation step during which artifacts can accidently be formed is sample extraction. In untargeted plant metabolomics, methanol-water mixtures with or without acidification (e.g., formic or acetic acid) have become popular extraction solvents [4], since they allow extracting a wide range of metabolites [1]. Artifacts can originate from various processes including formation of hemiaminals via N-oxide intermediates out of metastable natural constituents as for example alkaloids [5]. However, the most probable route of artifact formation is via reaction with solvent molecules. As reviewed by Maltese et al. [5], solvents (like methanol (MeOH)) can react with sample constituents, thereby forming so-called “solvent artifacts.” For example, methanol can cause the formation of a multitude of chemical derivatives such as esters from carboxylic groups (e.g., in fatty acids), acetals from hemiacetals, ethers from hydroxyl groups, transesterification [5], Michael addition to α,β-unsaturated carbonyls [6] or formation of α,α-methoxyamines from hemiaminals [7] and other solvent artifacts. Some of these reactions are promoted by acidic conditions, which may be provided by acidification of the extraction solvent.

Several studies describe the formation of solvent artifacts [6,8,9,10,11]. Brondz et al. [8] reported two artifacts (infractine and 6-hydroxyinfractine) in the inedible basidiomycete mushroom *Cortinarius infractus*, which were formed from β-carboline-1-propionic acid after extraction with methanol. An example of a substance class, which may also be methylated during sample preparation, are withanolides, which are a group of C-28 steroidal lactones [6]. Gu et al. [6] showed that solvent artifacts were formed via a Michael addition of methanol to isocarpalactone A and -B during extraction and purification of tomatillo plants (*Physalis philadelphica*). Interestingly, methanol can also lead to artifacts during RP-chromatography. Perry et al. [9] found two methanol-derived artifact compounds from clavigerin B and -C in *Lepidolaena clavigera*, which have been formed during RP-chromatography rather than during extraction, which did not use methanol.

Certainly, methylation is a process that occurs also frequently in nature. It is commonly known that methyltransferases cause transmethylation by transferring a methyl group from S-adenosylmethionine, the active form of methionine, to a nitrogen, oxygen, sulphur or another atom of an acceptor molecule [12]. For example, ferulic acid is synthesized from caffeic acid in a transmethylation reaction and chemical reactivity of phenolic groups can be controlled by the plant through transmethylation [12]. Since methyl-groups are very common in nature [5], it is very difficult to differentiate biologically methylated compounds from chemical such as solvent artifacts. Moreover, enzymatic reactions can lead to the formation of methanol-derived solvent artifacts, as shown by Stemmler et al. [11], who reported a C-terminally methylated neuropeptide in the lobster *Homarus americanus* as a result of an enzymatic reaction after peptide truncation in the presence of methanol.

From the above it can be concluded that especially in untargeted metabolomics studies, where the majority of the detected metabolites are unknown prior to analysis, it is important to recognize such solvent artifacts. Otherwise incorrect biological conclusions could be drawn. While contaminants, which originate from releasing agents, plastic softeners or impurities in the extraction solvent, can easily be detected by the use of water- or solvent-blank samples [2] or by the use of fully-^13^C-labeled sample material [13,14,15,16,17], solvent artifacts newly formed from sample metabolites are more complicated to detect. Such solvent artifacts can be detected using a SIL approach, which specifically labels all newly formed artifact compounds in the samples. Since most stable isotopically labeled compounds form highly characteristic isotopolog patterns in the raw LC-HRMS data, it is also possible to detect them with automated software tools.

The goal of this study is to evaluate whether the extraction of wheat leaves with commonly used methanol-containing extraction mixtures will lead to solvent derived artifacts. Acidified and non-acidified extraction solvents were compared with respect to the extent of artifact formation. Additionally, storage of samples at 10 °C (a typical temperature of an HPLC autosampler unit) for several days was tested for both the number and intensity of solvent artifacts. To the best of our knowledge the presented study is the first to evaluate the extent of solvent artifact formation in a commonly applied untargeted metabolomics workflow.

## 2. Results

### 2.1. Data Structure and Overview of Results

**Figure 1:**
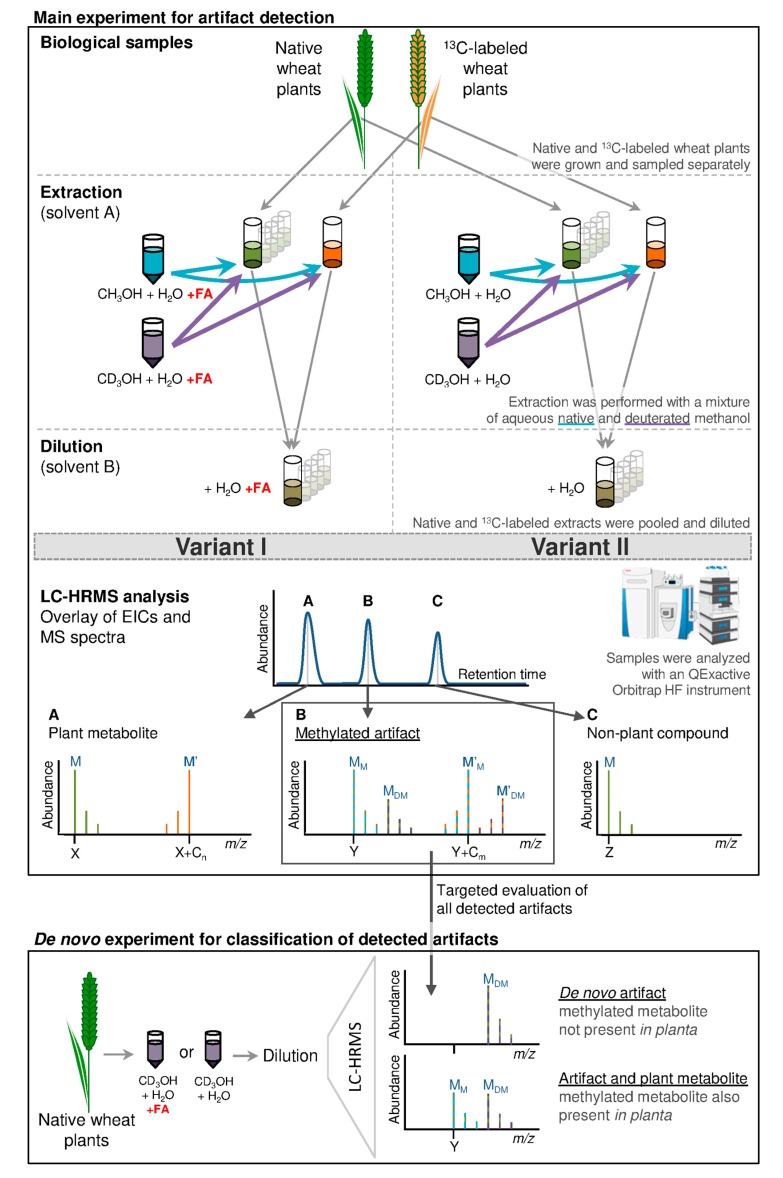
Overview of the experimental procedure and expected results for artifact compounds, plant metabolites and contaminants. Native and fully ^13^C labeled wheat leaves were extracted with mixtures of native and deuterated aqueous methanol (3:1 (*v/v*)) acidified with 0.1% formic acid (FA) for variant I and without the addition FA for variant II. The resulting extracts were combined and diluted with water (for variant I with FA) to obtain a final ratio of methanol/water of 1:1 (*v/v*). Samples were analyzed with LC-HRMS and an example chromatogram is shown. Schematically, the mass spectrum **A** represents a plant metabolite showing the characteristic isotope pattern originating from a native (M) and uniformly labeled plant leaf metabolite (M´). No additional deuterium containing isotopolog peaks are present. Mass spectrum **B** represents a methylated artifact where M_M_ represents the artifact formed from the reaction of the native plant metabolite with native methanol. The mass of M_M_ is increased by +14 or +32 compared to its educt (such as M in spectrum **A**); M_DM_ represents the deuterated methylated artifact (which originates from the reaction of M with CD_3_OH; mass increase of +17 or +35 compared to M); M’_M_ and M’_DM_ refer to the corresponding artifact analogs of the fully-^13^C-labeled plant metabolite M’. Mass spectrum **C** illustrates an unspecific signal not deriving from the plant, as no fully-^13^C-labeled peaks are present. Every single artifact served as a putative target in the *de novo* experiment which was used for classification of *de novo* artifacts. For this, native wheat leaf material was extracted with aqueous deuterated methanol (with and without the addition of FA) only. For artifacts classified as *de novo* M_M_ (shown in mass spectrum **B**) cannot be found after extraction and are therefore considered to be newly formed. If M_M_ was present in the candidate spectra, the methylated metabolite had already been present in the analyzed sample prior to analysis and a proportion thereof was additionally formed during metabolite extraction.

This study aimed to evaluate the extent of solvent artifact formation during extraction with aqueous methanol (MeOH) and subsequent short-term storage. Two different extraction solvents were investigated, for which artifact formation will be presented comparatively: (I) acidified MeOH/H_2_O (3:1 (*v/v*), 0.1% formic acid (FA)) and (II) MeOH/H_2_O (3:1 (*v/v*)). MeOH used for extraction in this study consisted of a mixture of 50% native (CH_3_OH) and 50% deuterated (CD_3_OH) MeOH. As has been described earlier the pH-value of the two extraction mixtures/extracts have been pH 4.8 and pH 7.5 respectively [4]. In the following, the two extraction mixtures will be indicated as variant I (vI, extraction with FA) and variant II (vII, extraction with pure aqueous methanol) (Figure 1).

Solvent artifacts are products formed from derivatization of a plant constituent with the extraction solvent. In case of methanol, this would lead to the formal addition of CH_2_ (e.g., esterification of a carboxylic group) or CH_3_OH (e.g., in a Michael addition to an α,β-unsaturated carbonyl compound) and thus to a chemically different and new compound with an increased mass of +14 or +32 Da compared to its educt respectively. In typical studies, where only native MeOH is employed as extraction solvent, such artifacts cannot be differentiated from true biological methylated metabolites. As a consequence, this study used a mix of native and D_3_-labeled MeOH for metabolite extraction. Due to the higher mass of CD_3_OH (in comparison to CH_3_OH), the mass of the newly formed artifact compound will be increased by +17 or +35 Da, resulting in a mass difference between the native and the D_3_-labeled artifact of +3 Da. Such a mass difference as well as the isotopolog pattern of respective compounds is unique and can therefore be efficiently used for artifact detection. Extracts were analyzed by LC-HRMS and data processing was performed with a customized version of the in-house developed MetExtract II software [18]. The software was used to automatically screen for pairs of native (^12^C) and fully-^13^C-labeled artifacts (M_M_ and M’_M_—Figure 1) and their respective isotopologs (M_M_ + 1 and M’_M_ − 1). If these isotopologs were found, the presence of corresponding peaks, with mass increments of +3 Da was also verified. The latter mass peaks constitute of the deuterated methylated native and fully-^13^C-labeled artifact (M_DM_ and M’_DM_) respectively. If all those isotopologs were detected, the respective compounds were considered to be artifacts formed during metabolite extraction, or subsequent storage with methanol. Artifact features (consisting of e.g., different ion species belonging to the same substance—e.g., [M + H]^+^ and [M − H]^−^) were grouped to artifact metabolites, as described by Bueschl et al. [18]. In the following, results will deal with artifact metabolites, unless indicated differently. For statistical data evaluation with R software [19], which was performed after manual verification of all artifact features with Thermo Xcalibur, the most abundant artifact features were chosen to represent respective artifact metabolites.

Nomenclature for samples: 0 dpe–10 dpe represents the time point of measurement after metabolite extraction (dpe = day post extraction, 0 dpe = immediate measurement after extraction); 10 °C, −20 °C or −80 °C specify the respective storage temperature; I or II indicates extraction with (variant I, +FA) or without (variant II, −FA) addition of 0.1% (*v/v*) formic acid. Artifacts that were not present in the original leaf metabolome were classified as *de novo* forms, while artifact compounds that were formed during metabolite extraction or sample storage but that were also already present in the plant leaves (i.e., endogenous metabolites) and were thus present as M_M_ as well as M_DM_ forms in the samples of the *de novo* experiment, were not annotated as *de novo* forms.

Variant I and II extracts were used for artifact detection. In total 88 solvent artifacts, which are represented by 177 artifact features (listed in detail in Table S1—supplementary material) were detected. The total number of detected plant metabolites was obtained from samples extracted as described by Bueschl et al. [13] (not shown in the workflow in Figure 1; see Chapter 4.3) and varied between 1028 and 1149 in samples extracted with acidified methanol, respectively 1046 and 1148 in samples extracted with pure aqueous methanol.

**Figure 2:**
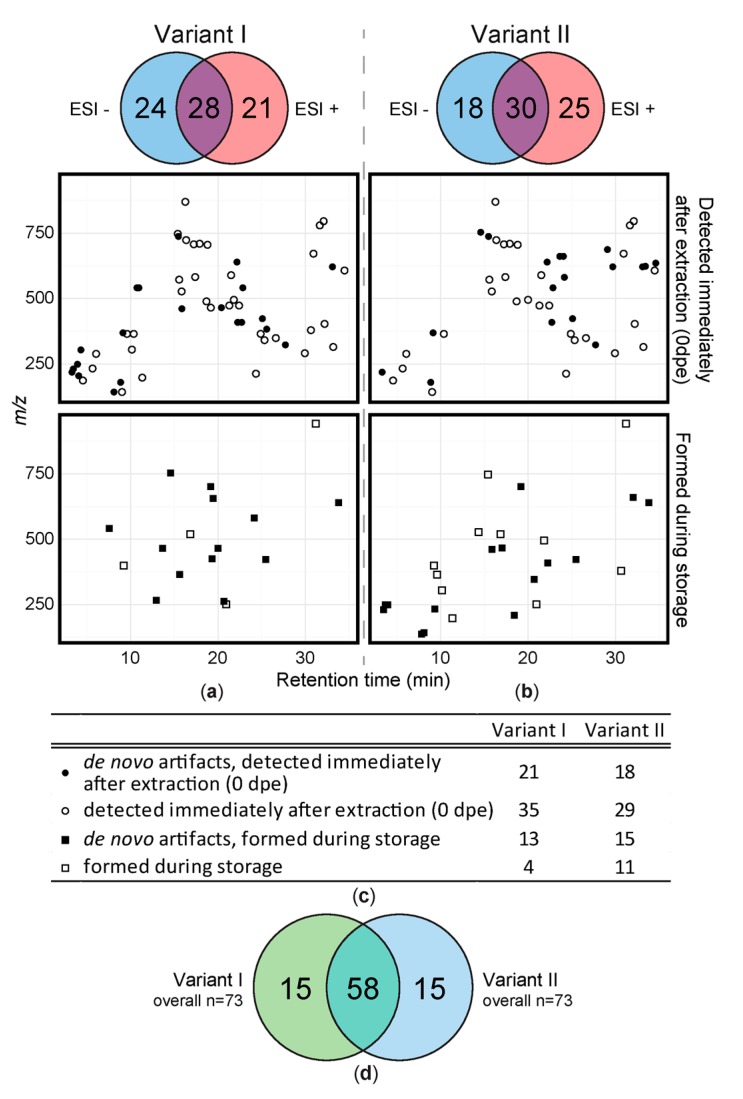
Overview of all detected solvent artifacts. 2D-plots of artifacts detected immediately after extraction and formed during storage. (**a**) acidified extraction mixture and (**b**) extraction mixture without acidification. Black symbols illustrate MeOH derived derivatives that were not present in the original leaf metabolome (*de novo*). White symbols: relative amount changed/increased due to reaction of native wheat constituents during extraction or storage. Circles represent artifacts formed during sample extraction, squares refer to artifacts that were detected during storage for up to 10 days. The Venn diagrams above the 2D-plots show polarity modes, in which respective numbers of artifacts were detected. (**c**) gives an overview of the number of metabolites detected directly after extraction and/or *de novo* artifacts. (**d**) number of artifact compounds detected in variant I (green) and variant II (blue).

About 8% of all metabolites were assigned to be solvent artifacts under the tested conditions. Figure 2 shows all detected artifact compounds in the form of a two-dimensional feature map. The detected artifacts are distributed throughout the entire chromatogram and *m/z* scan range and no accumulation in particular parts of the plot is evident. More precisely, artifacts with masses from *m/z* 138 to 941 and different polarity (based on chromatographic retention) were detected.

Potential educts (i.e., natural precursors) of the detected artifact compounds were annotated using an in-house compiled list of wheat metabolites collected from literature and the PlantCyc database. For calculating the putative educt’s mass both artifact variants (addition of CH_2_ or CH_3_OH) were considered since the type of chemical reaction leading to the formation of the respective artifact was not known. The masses of the putative educts were then compared to the database entries (maximum *m/z* difference: ±3 ppm). Any educt annotated with a database hit was subject to a manual EIC inspection. Any chromatographic peak present in the respective EIC that eluted earlier than the methanol-derived artifact peak was considered as a putative precursor substance. However, in most cases this resulted in more than one educt candidate. An additional problem in assigning the natural precursors to the measured artifacts was attributed to the fact that the extend of the transformation from the educt to the artifact was unknown. In case of complete methylation, the precursor would not have been present in the chromatograms at all. A list of all annotated and identified substances is provided in Table S1. We were able to identify two artifacts using authentic reference standards and to annotate another 13 artifacts with database hits. The annotated compounds included organic acids (e.g., fatty acids and oxygenated derivatives, citric acid, aconitic acid), flavonoids and carotenoids but also the chlorophyll break down product pheophorbide was found, showing that multiple substance classes were potential targets of chemical reactions with the solvent. 

A total of 73 artifacts were detected in the sample extracts of variant I (acidified extraction mixture) when considering all storage conditions and time periods (Figure 2a). For variant I, 28 artifacts (38%) were detected in both ionization modes while 21 (29%) were only detected in positive and 24 (33%) in negative mode respectively. 34 of the 73 artifacts were classified as having been formed *de novo* during extraction or storage and thus are not part of the original sample metabolome. Variant II sample extracts also showed 73 artifacts (Figure 2b) of which 33 were not present prior to extraction and therefore were presumably formed by reaction of native leaf constituents with methanol during extraction or storage (= *de novo*). 30 of those artifacts (41%) were detected in both ionization modes and 25 (34%) respectively 18 (25%) were only detected in either positive or negative ionization mode. Although the number of artifacts detected in variant I and II extracts is the same, their chemical identities are different to a considerable extent. While 58 of all artifacts, were detected with both extraction methods, 15 were only detected in either of the variant I and variant II samples respectively (Figure 2d).

### 2.2. Short Term Storage of Sample Extracts

**Figure 3:**
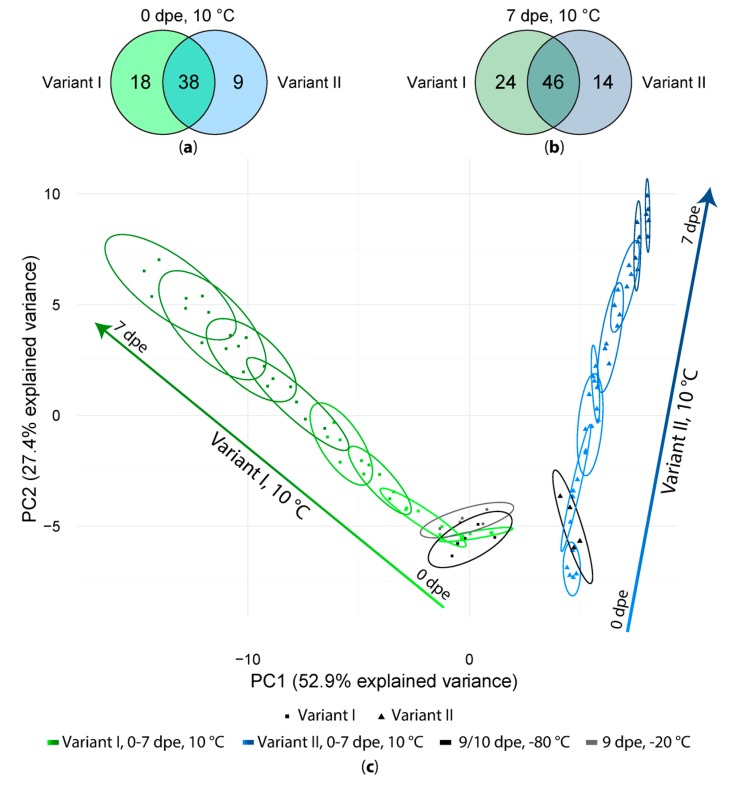
(**a**) Number of artifact compounds detected in freshly extracted samples (0 dpe) in variant I and variant II; (**b**) Same comparison as in (**a**) but for the samples measured after one week (7 dpe); (**c**) PCA score plot of all variant I and II samples.

After artifact compound detection, we tested the effect of long measurement sequences on the number and intensity of the extraction artifacts by storage of extracts at 10 °C, −20 °C and −80 °C and daily measurements of sample extracts over the period of 7 days. In general, 56 (variant I) and 47 (variant II) artifacts were present already in the first measurements (0 dpe), of which 38 were present regardless of whether formic acid was added during the extraction step or not. Additionally, 18 artifacts were detected only in the samples of variant I and 9 were detected only in the samples of variant II (Figure 3a). In general, the number of artifact compounds increased while the samples were stored at 10 °C for one week (Figure 3b) from 56 to 70 in variant I- and from 47 to 60 in variant II-sample extracts, out of which 24 (vI) respectively 14 (vII) artifacts have only been found in one of the tested variants. All experimental variants and detected artifact compounds obtained with one of the extraction mixtures were then subjected to a principal component analysis (PCA, Figure 3c). As can be seen in the PCA scores plot the samples extracted with and without FA are clearly separated from each other whereas samples of variant I are distributed along PC1 as well as along PC2. Samples extracted without FA (variant II) are mainly distributed along PC2.

Interestingly, the samples of both variants I and II stored at −20 or −80 °C but measured at 9 and 10 dpe are closest to the samples stored at 10 °C and measured immediately after extraction (0 dpe, 10 °C). Moreover, the samples measured after one week (7 dpe) are clearly separated from those of their respective freshly prepared extracts (0 dpe). In general, sample extracts of one time point tend to overlap with the previous and following time point (±1 day) but are separated from samples extracts with time points differing by at least 2 days. This indicates that storage at 10 °C for several days resulted in a continuous change of the abundance patterns of the detected artifact compounds over storage time.

### 2.3. Intensities of Solvent Artifacts

**Figure 4:**
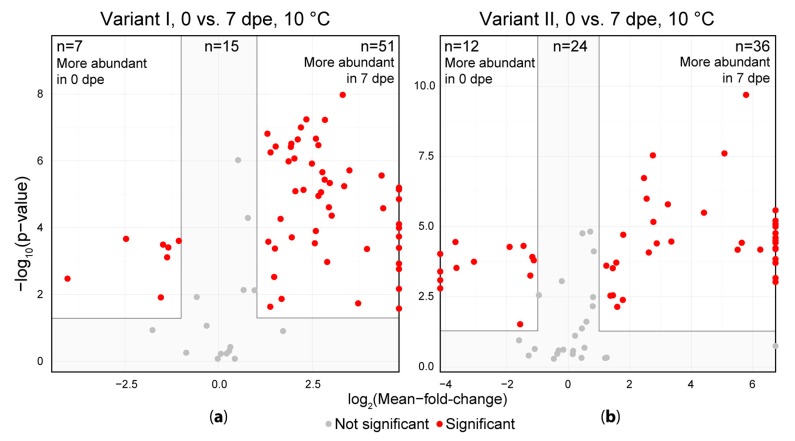
Comparison of sample extracts measured immediately after extraction (0 dpe) and after one week of storage in the auto sample unit (7 dpe). (**a**) shows the acidified extraction solvent (variant I) and (**b**) shows the samples extracted without the addition of formic acid (variant II). Each dot represents one artifact compound. Artifacts depicted on the left or right vertical line (border of the volcano plot) indicate, that the respective artifact was only detected in either one the two groups and thus the respective fold is infinite.

The detected artifact compound abundances were then subjected to a univariate significance testing using the immediate measurement (0 dpe) and measurement after one week (7 dpe) to investigate the extent of the change in their intensity during storage of extracts (Figure 4). Of the artifacts in variant I, 51 showed significantly higher abundances after a storage duration of one week (7 dpe) at 10 °C compared to the freshly obtained extracts (0 dpe). Only 7 artifacts were more abundant on 0 dpe compared to 7 dpe, while 15 artifacts did not show a significant variation between the samples of 0 and 7 dpe. In the samples of variant II, 36 artifacts significantly increased in intensity after storage for 1 week at 10 °C compared to the freshly obtained extracts and only 12 artifacts decreased in intensity during the same storage duration while 24 artifacts did not change significantly. For both variants, some metabolites were solely present in the samples measured after one week of storage at 10 °C indicating that these compounds were not formed during extraction but rather during subsequent storage of the extracts.

Table 1 provides an overview of artifact abundances in the sample extracts and to evaluate their intensity increase or decrease over storage time, a color coded (logarithmic color scale) table was generated. It provides a quick overview of the change of artifact intensities during sample extract storage. The abundances of the majority of the detected artifacts increased over the storage duration (e.g., artifacts 4, 7, 8, 12, 14, 15). In addition, the abundances of the artifacts in the extracts stored for 9 days at −20 °C or −80 °C were in most cases similar to the levels observed in the immediately measured extracts (e.g., artifacts 1, 4, 7, 8). The abundance of the artifact representing methylated citric acid (artifact 5) increased during the storage over one week at 10 °C after extraction with acidified methanol, while the abundance of the artifact did not change in the samples stored at −20 or −80 °C. Interestingly, the methylated citric acid was not detected in samples extracted without the addition of formic acid. Another identified methylated artifact (methylated aconitic acid, artifact 7) had a similar abundance trend and also was more abundant after 1 week of storage of the extract at 10 °C. Additionally, it was also detected in variant II samples.

For all artifact compounds the distribution of the abundances at the respective measurement time points was evaluated and the abundances were then compared to the abundance levels of all other detected plant metabolites (supplementary Figure S1). The intensities of the artifacts ranged from 2 × 10^2^ to 4 × 10^8^ and are similarly distributed as for all plant metabolites (including the artifacts). Moreover, the average abundance was approximately the same for both artifacts and plant metabolites (~10^5^). While the abundance values of the total set of plant metabolites remained constant during the one-week storage at 10 °C, the intensity levels of the detected methanol artifact compounds tended to increase over time (density plots in Figure S1b). In agreement with Figure 4 the intensity levels of the artifacts and especially the *de novo* artifacts in the acidified extracts (variant I) increased to a greater extent compared to the same artifacts detected in the samples of variant II. This indicates that (at least) with respect to the artifact compounds, acidified extracts (variant I) were less stable compared to those obtained after extraction with non-acidified aqueous methanol.

## 3. Discussion

### 3.1. Discussion of General Results

In untargeted metabolomics, the majority of the detected metabolites are unknown prior to analysis and most of them also remain unknown or only annotated after data evaluation [13]. In general, metabolomics studies compare different experimental conditions (e.g., treatment vs. control groups) and then compare the observed metabolite levels between the tested conditions. To detect the respective metabolites in the analytical data, different software tools are used that automatically detect all compounds and report them in a comprehensive form for further processing (e.g., statistical analysis). Hence, any non-sample related compounds (e.g., softeners or polymer degradation products, solvent contaminants, as well as chemical artifacts caused by chemical reactions of sample compounds with the solvent during extraction or storage) are also automatically detected and reported as putative metabolites of the biological sample under investigation. However, if not recognized and removed, these process artifacts can lead to incorrect biological conclusions. The aim of this study was to answer the question, if solvent artifacts are formed and to which extent they are present in wheat leaf samples when typically used extraction solvents and protocols are applied [1,4].

We first investigated the total number of metabolites (including putative solvent derived extraction artifacts). Here we detected 1149 plant-leaf-specific metabolites after extraction with acidified aqueous methanol and up to 1148 metabolites upon extraction with pure aqueous methanol. Next, we applied our SIL approach for the detection of artifact compounds and detected a total of 88 artifacts under the tested conditions, which corresponds to approximately 8% of all metabolites. Artifacts were formed regardless of mass and polarity of the respective plant constituents (i.e., both, polar and apolar compounds formed solvent artifacts) as artifacts were detected throughout the entire chromatogram. Moreover, artifact compounds were found in both ESI ionization modes and with both extraction mixtures (vI and vII), indicating that many chemical compound classes were effected by reaction with the extraction solvent. In good agreement with this and the fact that methanol can react with various chemical functions like carboxylic groups, aldehydes, ketones, hydroxyl groups [5] or α,β-unsaturated carbonyls [6] we identified citric acid and cis-aconitic acid to be methylated through the extraction process and also fatty acids were annotated in our experiment (Table S1, supplementary material). With respect to carboxylic acids Rajniak et al. [10] analyzed indole-3-carboxylic acid methyl ester that was found in *Arabidopsis* extracts and confirmed it to be a solvent artifact of indole-3-carboxylic acid by extraction with deuterated methanol. We did not find this methyl ester nor its chemical precursor indole-3-carboxylic acid in our plant extracts. Instead we found artifacts with educts belonging to various other substance classes such as flavonoids and carotenoids in our samples. We found that especially typical secondary metabolites such as flavonoids or carotenoids were targets of methylation under the tested conditions. The tendency of these types of compounds to react with methanol therefore has to be considered as it may have a detrimental effect on any metabolomics study considering these substance classes or other phenols and polyenes respectively. Moreover, the uniform distribution of the detected artifact compounds in the entire chromatogram (Figure 2) suggests that numerous different substance classes, which can be of interest in (untargeted) metabolomics studies, can be targets of meth(ox)ylation.

While the total number of 73 artifacts did not differ between the two extraction variants (and thus extraction pH of ~5 and 7.5 respectively [4]), we observed that the abundances of artifacts increased to a greater extent in acidified methanol compared to the neutral extraction conditions. Moreover, the relative change in abundance of both types of metabolites, natural precursors as well as the resulting artifacts has also to be considered since this may significantly influence the outcome of a metabolomics study. Therefore, we tried to distinguish between two forms of solvent artifacts: (1) *De novo* artifacts that were formed during extraction or storage and have not been in the original sample before extraction with methanol and (2) compounds which already had been constituents of the sample before extraction (true plant-leaf metabolites) but were additionally found to be formed during metabolite extraction or storage to a certain extent. This fact has to be taken into consideration when relative metabolite levels are compared between different experimental conditions, which is often the case in untargeted metabolomics-studies. *De novo* artifacts will lead to wrong metabolite annotations and wrong conclusions with respect to metabolic composition. In addition, *de novo* formed artifacts may also lead to incorrect biological interpretations. The second type of artifacts does rather bias metabolite abundance and depending in the relative extent of artifact formation may mask true metabolic changes. Moreover, in case of quantitative reaction with methanol the respective native precursor constituents may be missed completely. In our experiment, we found that 47 of the 88 (53%) artifacts were formed *de novo*, during extraction or storage of extracts, which corresponds to a total of 4% of the total number of detected metabolites, demonstrating that metabolite reaction with methanol could have a considerable influence upon the evaluation and interpretation of untargeted metabolomics studies.

### 3.2. Short Term Storage of Sample Extracts

Especially large metabolomics studies with many experimental variants and biological or technical replicates and QC samples might require long measurement sequences. Then samples are typically placed in the autosampler unit of the HPLC instrument for several days before being measured. Our study showed that already upon immediate sample measurement, a considerable number of artifacts can be detected and that the number of solvent artifacts even increased continuously with storage time (we have tested up to 1 week) at 10 °C for both acidified and non-acidified sample extracts. This leads to the conclusion that methanolic sample extracts change when stored at 10 °C for several days and that they are not stable in respect to solvent artifact abundance levels. The fact that artifact abundances are increasing and new artifacts are forming (sometimes out of other artifacts) in a relatively short time period can lead to inconsistent findings for replicates of a metabolomics experiment measured over several days. Thus, to avoid this problem, sample extracts should either be measured as soon as possible after extraction or be frozen until being measured. However, it should be noted that in this case the effect of freezing and thawing on the global metabolome also has to be considered. For example, studies by Eilertsen et al. [20], who used direct infusion HRMS of microalgae extracts have shown that one freeze/thaw cycle (at −78 °C) caused a loss of 10% of the signals. After a second freeze/thaw cycle a total of 20% of the signals were lost compared to immediate measurement of fresh samples. Moreover, Pinto et al. [21] observed that lipid content in samples also has an impact on the stability of human plasma samples during freezing and thawing. The authors therefore also recommend to not have more than three freeze/thaw cycles for human plasma samples for NMR based metabolomics. The effect of freezing and thawing in plant extracts combined with untargeted LC-HRMS should also be evaluated in the future but was beyond the scope of this study.

### 3.3. Intensities of Solvent Artifacts

As already mentioned above, the intensity of most solvent artifact LC-HRMS peaks, increased with storage duration. This effect was more distinct for the acidified extracts compared to non-acidified aqueous methanol extracts. Our results show, that especially at 10 °C the levels of solvent artifacts increased substantially, whereas at −20 and −80 °C they did practically not change over a storage period of 10 days. Since the general trend of an increase in artifact intensity levels was only observed for artifacts and not for all of the plant metabolites, we conclude that this increase was not caused by an instrument sensitivity drift during the measurement period of 11 days. Interestingly we also observed some artifacts that had decreased intensity levels after 1 week of storage at 10 °C. Those artifact substances were probably further transformed to other reaction products, or the reverse reaction might also have occurred. In addition to the general change in abundance of artifacts, the relative change due to artifact formation of those metabolites that occur in both non-methylated and methylated forms in the experimental samples is also of interest (see Section 3.1). For example, if 90% of the measured intensity value of an artifact is based on the reaction with the solvent, artifact formation would have a rather big influence on a study compared to a metabolite whose abundance is only increased by 10% by artifact formation. For calculation of the relative rate of artifact formation, the natural precursor metabolite of which the detected artifact was formed needs to be known. However, this proved to be difficult to investigate. In our study, we were able to identify a few artifacts and their putative educts by methylation of the reference standard with native and deuterated methanol in pure solvents (see Section 4.4). For this, the accurate mass of the putative educt was calculated by subtracting the exact mass of CH_2_ respectively CH_3_OH from the accurate mass of the artifact. Methylation or methoxylation by reaction with methanol should make artifacts more apolar in respect to their educts and therefore the educts were expected to elute earlier than their corresponding artifact. However, in our study for the majority of the detected artifacts multiple peaks fulfilled those criteria making it difficult to properly select the candidate educt. In addition, in many cases both the EIC traces of *m/z* (M_M_) minus *m/z* (CH_2_) and *m/z* (M_M_) minus *m/z* (CH_3_OH) showed chromatographic peaks in the LC-HRMS data. Thus, it was not possible to unambiguously classify the artifact formation to be caused by methylation or methoxylation respectively. Without any knowledge about the identity of the methylated peak, it was impossible for us to determine the correct educt and therefore this needs to be further investigated (e.g., by MS/MS experiments). On average, the abundances (distribution and mean values) of the detected artifacts were comparable to those of all (other) plant metabolites (Figure S1). From this finding, it can be expected that methanol-derived artifacts can be found in extracts of experimental samples across the whole range of measured feature intensities.

## 4. Materials and Methods

### 4.1. Chemicals

Methanol (LC-MS Chromasolv™, HPLC grade) was purchased from Riedel de Haen, Honeywell (Seelze, Germany); deuterated methanol (<0.1% water, 99.5% D_3_) from Eurisotop (St-Aubin, Cedex, France); acetonitrile HiPerSolv HPLC-Grade from VWR Chemicals (Vienna, Austria); ELGA water was obtained from an ELGA Purelab Ultra-AN-MK2 system—Veolia Water (Vienna, Austria). Formic acid (MS grade) was purchased from Sigma-Aldrich (Vienna, Austria).

The following reference standards were intentionally methylated in order to identify artifacts: cis-aconitic acid (Aldrich, Vienna, Austria), citric acid (Merck, Darmstadt, Germany).

### 4.2. Cultivation and Sampling of Plant Material

Native and uniformly-^13^C-labeled wheat plants (*Triticum aestivum*, cultivar “CM-82036”, 98.6% ^13^C) were cultivated in a closed growth chamber under controlled atmospheric conditions. Therefore, temperatures were varied between 14 and 20 °C and day-night-regime between 12/12 and 14/10 h. CO_2_-content was held at 400 ppm and O_2_-content at 20%. A slight overpressure of 10 mbar was applied, to prevent ambient air from entering the growth chamber. This was especially important for full-^13^C-labeling, where ^12^CO_2_ was replaced by ^13^CO_2_. Plants were grown in hydroponics with a nutrient solution adapted from Hoagland et al. [22]. They were irrigated one to three times a week, depending on their nutrient solution uptake. Upon flowering, plants were harvested and frozen immediately with liquid nitrogen to stop any metabolic activity. Fully-^13^C-labeled samples were freeze-dried (Labconco FreeZone 6Plus; Labconco, Kansas City, MO, USA). All samples were stored upon −80 °C until extraction.

### 4.3. Sample Preparation and Extraction of Plant Material

A schematic overview of the extraction procedure is given in Figure 1. Many untargeted metabolomics studies deal with analysis of leaves of different plants [4] and therefore, wheat leaves were chosen for this study. (Freeze dried) leaf samples were ground to a fine powder with a ball mill (MM400—Retsch, Haan, Germany) under cooling with liquid nitrogen. Native wheat samples were pooled and homogenized thoroughly. 100 ± 2 mg of non-freeze-dried samples respectively 30 ± 1 mg of freeze-dried samples were weighed into a 2 mL tube. Then 70 µL of water was added to freeze-dried samples, to replace the water, which was removed upon freeze-drying. Immediately after this, extraction was carried out based on the extraction procedure described by Bueschl et al. [13].

In variant I (5 replicates), two separate extractions were performed, where 500 µL of extraction solvent A_1+FA_ (C**H_3_**OH:H_2_O 3:1 (*v/v*) containing 0.1% FA (*v/v*)) and 500 µL of extraction solvent A_2+FA_ (C**D_3_**OH:H_2_O 3:1 (*v/v*) containing 0.1% FA (*v/v*)) were added to either native or fully-^13^C-labeled samples respectively. Samples were vortexed for 10 s and then sonicated for 15 min. After centrifugation for 10 min at 14,000 rpm at 4 °C, supernatants of native and of fully-^13^C-labeled samples were mixed 1:1 (*v/v*) and subsequently diluted with solvent B_+FA_ (H_2_O with 0.1% FA (*v/v*)) to get a final MeOH:H_2_O ratio of 1:1 (*v/v*).

To test whether the extraction with non-acidified solvent would influence the number and type of artifact compounds, the above described extraction protocol was also applied without the addition of FA to the extraction solvent (variant II). Extraction solvents were replaced by solvent A_1_ (C**H_3_**OH:H_2_O 3:1 (*v/v*)), A_2_ (C**D_3_**OH:H_2_O 3:1 (*v/v*)) and dilution of raw extracts was carried out by solvent B (pure H_2_O).

Additionally, in the *de novo* experiment native wheat samples were extracted with 1 mL of deuterated aqueous methanol only (solvent A_2+FA_ and A_2_ respectively) following the same extraction procedure to test for *de novo* formed artifacts.

For detection of the total numbers of metabolites an additional experiment was carried out where plant samples were extracted in the same way with 1 mL native methanol only (native and fully-^13^C-labeled samples; solvent A_1+FA_ and A_1_ respectively) according to the procedure described by Bueschl et al. [13].

### 4.4. Preparation of Methylated Citric Acid and Cis-Aconitic Acid

For confirmation of formation and verification of artifact identity, citric acid as well as cis-aconitic acid have been dissolved in the extraction mixtures of native and deuterated methanol (solvent A_1+FA_ and A_2+FA_ respective A_1_ and A_2_). Subsequently, they were measured as described below.

### 4.5. Influence of Storage Time and Conditions

Sample extracts were split in several aliquots and stored at 10 °C (which is the temperature of the autosampler unit), −20 °C or −80 °C for up to 10 days. Sample extracts stored at 10 °C were analyzed immediately after extraction (0 dpe) as well as daily throughout the following 7 days (1–7 dpe). Additionally, 5 replicates of sample extracts of variant I were stored at −20 °C and −80 °C respectively for 9 days (9 dpe) and replicates of sample extracts of variant II were stored at −80 °C for 10 days (10 dpe).

### 4.6. LC-HRMS Analysis of Wheat Leaf Samples

A UHPLC-system (Vanquish—Thermo Fisher Scientific, San Jose, CA, USA) with a C_18_-column (X-Bridge, 150 × 2.1 mm i.d., 3.5 µm particle size—Waters, Milford, MA, USA) equipped with a pre-column (C_18_ 4 × 3 mm i.d., Security Guard Cartridge, Phenomenex, Torrance, CA, USA) was used for chromatographic separation. As described by Bueschl et al. [13], two eluents, H_2_O with 0.1% FA (*v/v*) (eluent A) and MeOH with 0.1% FA (*v/v*) (eluent B), were applied for gradient elution. The 45 min long method started with 10% B which was held constant for 2 min. Then B increased linearly to 100% within 30 min. After that B was held constant at 100% for 5 min followed by equilibration of the column at 10% B for 8 min. During chromatographic separation, the flow rate was held constant at 250 µL/min and the column was thermostated to 25 °C.

Sample extracts were analyzed with an HESI-QExactive HF-Orbitrap-HRMS (Thermo Fisher Scientific, San Jose, CA, USA). The metabolites were ionized with fast polarity switching (+/− ionization mode) and analyzed in full scan mode. The scan range was set to *m/z* 100–1000 and the resolution was set to 120,000 at *m/z* 200. Instrument performance was monitored with a QC-mix containing 25 standard substances regularly injected within the measurement sequences.

### 4.7. Data Processing

LC-HRMS raw data files were converted to the mzXML format using ProteoWizard’s MSConvert software [23] (version 3.0.9322), [24] and subsequently processed either with the standard MetExtract II software [18] or a version custom-tailored for the specific characteristics of the deuterated meth(ox)ylated artifacts.

For data processing of the samples extracted in the additional experiment according to Bueschl et al. [13], the standard implementation of the MetExtract II software was used as described by Bueschl et al. [18]. Briefly summarized, the MetExtract-software searched for pairs of corresponding ^12^C- and ^13^C-signals in each MS-scan. Detected signal pairs were subsequently clustered according to their retention times and *m/z*-values. Then, EICs of the ^12^C- and ^13^C-form (M and M’, Figure 1a) were calculated for each of the obtained “*m/z*-bins.” Chromatographic peaks in both EICs were searched for but only those chromatographic peaks co-eluting in both EICs were further considered as true metabolite-derived features (i.e., metabolite ions) while any chromatographic peaks present in either one of the EICs which did not have a pendant in the respective other EIC were discarded as non-plant specific features. Different adducts (e.g., [M + H]^+^ and [M + Na]^+^) and in-source fragments (e.g., loss of H_2_O) of the same compounds were convoluted into metabolite groups using their highly similar retention times and chromatographic peak shapes. This resulted in a list of plant-derived metabolic features. Each feature was characterized by a unique ID, *m/z*, R_t_, number of C-atoms, charge state, ion species (if available) and EIC peak areas in all processed samples.

For the detection of meth(ox)ylated artifact compounds, the same MetExtract II software was customized to recognize the specific isotopolog patterns of native and uniformly ^13^C as well as native and D_3_-labeled compounds. This custom-tailored version was used to process the respective samples of the variants I and II as well as the *de novo* experiment. Similar to the standard version of the software tool, this implementation also searched for pairs of corresponding ^12^C- and ^13^C-signals (M_M_ and M’_M_—Figure 1b). However, it additionally also searched for the mass increment of +3.0188 Da (mass of 3 × D—mass of 3 × ^1^H) for both, the native as well as the ^13^C-labeled metabolite form (M_DM_ and M’_DM_). After peak quartet detection, all *m/z* values were clustered according to their retention times and *m/z*-values. Then, EICs of the ^12^C- and ^13^C-form were calculated for each of the obtained “*m/z*-bins” and inspected for chromatographic peaks co-eluting in both EICs. Additionally, the EICs of M_DM_ and M’_DM_ were also calculated and inspected for chromatographic peaks, which were allowed to show a minor chromatographic shift (max. 0–−7 scans), which is frequently observed in LC-HRMS measurement of deuterated compounds. Then, the detected artifact features were also merged into artifact compound groups, as described above.

The obtained list was manually verified by inspecting the raw data with Thermo Xcalibur software (Version 4.0.27.19). Only artifact metabolites, which were present in all 5 replicates in at least one group of extracts (e.g., vI, 1 dpe, 10 °C), were verified manually, all others were excluded. If M_M_, M_DM_, M’_M_ and M’_DM_ with the corresponding isotopologs were found, the respective feature was considered to be a solvent artifact. Additionally, the deuterated ions M_DM_ and M’_DM_ were not allowed to be present in the sample extract of the additional experiment, as there was no CD_3_OH added to the extraction solvent (see Section 4.3). If they were detected in the sample extracts of the additional experiment, they were considered false-positives of randomly co-eluting metabolites and removed from the data matrix.

### 4.8. Statistical Data Evaluation

Statistical data evaluation was carried out in the R environment [19] (version 3.1.0) and R-Studio (version 0.97.551, available online: https://www.rstudio.com, last accessed July 2017) using only the manually verified artifacts. The initial step of the statistical analysis was selecting one representative feature pair (*m/z* value of monoisotopic isotopolog, retention time, number of carbon atoms, charge state, ionization mode, adduct if annotated) per artifact group, which was the one with the maximum average abundance among all samples of the variants I and II. Any other feature pair also present in the feature groups were omitted. Moreover, only feature pairs detected in at least 4 out of 5 sample extracts of any experimental variant of vI or vII were used. This data matrix is referred to as D_c_ in the following.

#### 4.8.1. Selection of *De novo* Artifact Compounds

To distinguish artifact compounds already present in the biological samples from those that were solely formed during sample preparation with methanol or storage of extracts the samples of the *de novo* experiment were used. Any feature pair from D_c_ that was detected only as the deuterium-methylated artifact (M_DM_) (assigned as described above) but not as the methylated artifact (M_M_) in at least 12 of the 16 *de novo* experiment samples was assigned to be a *de novo* artifact formed by meth(ox)ylation (Figure 1). All other artifacts were classified as being derived from a reaction with methanol.

#### 4.8.2. Venn Diagrams

A feature pair was assigned to a specific experimental variant if it was detected in at least 4 of 5 replicates.

#### 4.8.3. Feature Map

The 2D-maps illustrating the detected artifacts used all feature pairs of D_c_ regardless in which of the experimental variants a feature pair was detected.

#### 4.8.4. Univariate Comparison between Metabolite Abundances of Two Variants

To compare two experimental variants of sample extracts, all feature pairs from D_c_ were used. For significance testing the two-sided *t*-test with a critical *p*-value of 0.05 was utilized. Additionally, a mean-fold-change between the average abundance of two variants of at least 2 or less than 1/2 was required for any feature pair to be assigned significantly different between the two tested extraction variants. The results of this univariate comparison and significance testing were illustrated in form of volcano plots with logarithmic scales.

#### 4.8.5. Abundance Histograms

The abundance histograms illustrate the distribution of peak areas of metabolite ions. They were calculated using the arithmetic mean values of EIC peak areas for the replicates of a particular experimental variant. The x-axis which illustrates peak area bins is shown as a 10-logarithmic scale and the abundances were sorted into 30 different bins.

#### 4.8.6. Principal Component Analysis (PCA)

For PCA all samples of the variants I and II and all feature pairs of D_c_ were used. Data pre-treatment comprised of replacing missing values by zero and auto-scaling of the abundances of the feature pairs (data matrix D_s_) [25] prior to calculating the PCA with the R-package ChemometricsWithR [26] (pp. 53–57). The ellipses were calculated with the co-variance matrices of the first and second principal components (PC1 and PC2) of the respective experimental variants using the R-package ellipses [27].

## 5. Conclusions

In this study two solvent mixtures commonly used in metabolomics experiments were used to evaluate the formation of methanol-derived meth(ox)ylation artifacts during sample extraction and storage of the generated extracts. Since methylated metabolites are frequently found as natural constituents of biological samples, this type of artifact is difficult to recognize by common untargeted metabolomics approaches. Here, the use of deuterated methanol and acidified deuterated methanol for extraction, subsequent LC-HRMS measurement and automated data processing by MetExtract II enabled for the first time the global unbiased assessment of artifacts which can be attributed to meth(ox)ylations of natural metabolites by the solvent methanol. At the example of wheat leaves we found that about 8% (*n* = 88, with 58 being common between two tested variants) of all detected leaf metabolites were artifacts, which were formed during sample extraction or storage of diluted sample extracts at 10 °C. Both primary and secondary metabolites of different chemical compound classes such as small carboxylic acids, fatty acids, phenols, carotenoids and pyrrols were among the (putatively) affected metabolites. Together with more than half of those artifacts having been formed by reaction with methanol *de novo* during extraction or storage and the fact that artifact abundances showed distributions similar to those of all other plant metabolites, our study demonstrates that great care must be taken when it comes to annotation and biological interpretation of methylated compounds. Moreover, we found that meth(ox)ylation continued during storage of diluted extracts in the LC-HRMS autosampler unit at 10 °C for one week, in particular under acidic conditions. This was not the case at −20 °C or −80 °C, thus sample extracts can be stored in the freezer to prevent further reactions of sample constituents with methanol. The large scale (tentative) identification of the methanol-derived artifacts was complicated by multiple putative precursor candidates and needs more detailed studies in the future. We hope however that the provided list of all detected artifact features can still be of general help for the reliable evaluation of other studies in the field of plant metabolomics.

## Figures and Tables

**Table 1:** Overview of all detected artifacts, illustrated by the most abundant feature per artifact compound. The complete table with all detected artifact features and additional information is provided in Table S1 (supplementary material). Mean peak areas (*n* = 5 replicates) of respective artifacts are shown in a logarithmically scaled color code. For calculation of mean peak area per artifact the respective metabolite compound ions had to be present in at least 4/5 replicates. If more than one ion was detected for an artifact, features belonging to the same artifact metabolite are indicated with _A, _B, …; **: identified by experimental methylation of the authentic reference standard with a mixture of native and deuterated methanol. *: substance class is probably known (for detailed information see Table S1).

ID		Methylated Form = Artifact									Variant I (+FA)																Variant II (−FA)											
											−20 °C	−80 °C			10 °C												−80 °C			10 °C								
											Storage Time [dpe] (Days Past Extraction)																											
		*m/z *of M_M_				R_t _[min]		*De novo*			9	9			0	1	2		3		4	5	6			7	10			0	1	2		3	4	5	6	7
1		[+]: 218.9587				3.30		x																														
2		[−]: 231.0509				3.45		x																														
3		[+]: 248.9693				3.65		x																														
4		[−]: 249.0614				3.93		x																														
5 **		[−]: 205.0353				4.07		x																														
6		[−]: 303.9520				4.32		x																														
7 **		[−]: 187.0246				4.55		×																														
8_B		[+]: 232.9745				5.65		x																														
9_A *		[−]: 143.0349				6.05																																
10		[−]: 541.1667				7.58																																
11 *		[+]: 138.0550				7.81																																
12_A*		[−]: 143.0349				8.10																																
13		[+]: 180.1019				8.86																																
14		[−]: 143.0348				9.02																																
15_C		[M + H]^+^: 369.1653				9.13																																
16		[+]: 399.1758				9.23																																
17 *		[+]: 234.1338				9.33																																
18_A		[M − H]^−^: 365.1352				9.59		x																														
19_B		[M + H]^+^: 305.1605				10.15																																
20_A		[M − H]^−^: 365.1351				10.36		x																														
21		[−]: 541.1668				10.73		x																														
22		[−]: 541.1666				10.95		x																														
23 *		[+]: 198.1124				11.34		x																														
24		[+]: 267.1203				12.93		x																														
25		[−]: 465.2332				13.67		x																														
26		[−]: 527.1873				14.34																																
27		[−]: 753.2617				14.57		x																														
28		[+]: 747.2841				15.40		x																														
29_A		[M − H]^−^: 737.3043				15.47		x																														
30		[−]: 572.2254				15.56																																
31		[−]: 365.1351				15.62		x																														
32		[−]: 527.1877				15.84																																
33_A		[−]: 461.2386				15.87		x																														
34		[−]: 869.3465				16.26		x																														
35_B		[M − H]^−^: 723.2886				16.36																																
36		[+]: 519.2415				16.83		x																														
37		[−]: 467.1012				17.03		x																														
38		[−]: 707.2937				17.23																																
39_C		[M − H]^−^: 737.2678				17.40		x																														
40_D		[M + H]^+^: 709.3071				17.90		x																														
41 *		[+]: 209.0811				18.40		x																														
42_C		[−]: 489.2337				18.68																																
43_B		[M − H]^−^: 705.2776				18.80		x																														
44		[−]: 701.2082				19.16		x																														
45		[−]: 465.1795				19.18																																
46		[+]: 425.2140				19.34		x																														
47		[−]: 655.3539				19.45																																
48		[+]: 495.2195				19.99		x																														
49		[−]: 465.1793				20.01		x																														
50		[−]: 465.1798				20.39																																
51		[+]: 263.1616				20.66		x																														
52		[−]: 347.1244				20.69																																
53_B		[+]: 251.1253				20.94		x																														
54_D		[−]: 473.2390				21.29		x																														
55		[−]: 589.2859				21.50		x																														
56_C		[M + H]^+^, [M + Na]^+^: 495.2196				21.81		x																														
57		[−]: 639.359				22.17																																
58_A		[M + Na]^+^: 409.2193				22.25																																
59_B		[−]: 473.2390				22.41																																
60		[+]: 409.2193				22.71																																
61_D *		[M + H]^+^: 541.1699				22.84																																
62		[+]: 661.2500				23.62																																
63		[+]: 661.2500				24.03		x																														
64_A *		[M − H]^−^: 581.1662				24.14																																
65_B *		[M + Na]+: 235.1303				24.35																																
66_C		[M + Na]^+^: 365.2296				24.88																																
67		[+]: 423.2348				25.08																																
68_A		[M − H]^−^: 341.233				25.32																																
69		[+]: 423.2349				25.48																																
70		[+]: 383.2193				25.57																																
71_B		[M + Na]^+^: 349.1982				26.61																																
72		[−]: 323.2227				27.71																																
73_B		[−]: 687.2312				29.07		x																														
74		[−]: 621.2721				29.68		x																														
75_B		[+]: 331.224				29.93		x																														
76		[+]: 379.2839				30.63																																
77		[+]: 671.4276				30.92		x																														
78		[+]: 941.5072				31.18																																
79_A		[+]: 779.4543				31.64		x																														
80		[+]: 659.2468				31.99																																
81		[+]: 403.2817				32.19		x																														
82_C		[M + Na]^+^, [M + CH_3_OH + H]^+^: 795.4858				32.08		x																														
83_B *		[M + Na]+: 315.2293				33.18		x																														
84_B *		[M + H]^+^: 621.2703				33.09		x																														
85_B		[M + H]^+^: 623.2858				33.41		x																														
86 *		[+]: 639.2806				33.79		x																														
87_B		[M + H]^+^: 607.2911				34.47		x																														
88		[−]: 635.2514				34.59																																
	Color code:			not present			<1×10^3^			1×10^3^–1×10^4^				1×10^4^–1×10^5^						1×10^5^–1×10^6^					1×10^6^–1×10^7^				1×10^7^–1×10^8^									

## Supplementary Materials

The following are available online at www.mdpi.com/2218-1989/8/1/1/s1, Table S1: Overview of all artifacts detected in the study; Figure S1: Abundance distribution of artifacts and plant metabolites.
