# Supplementary material for: Methanol Generates Numerous Artifacts during Sample Extraction and Storage of Extracts in Metabolomics Research
Source: Metabolites. 2017 Dec 22;8(1):1. doi: 10.3390/metabo8010001 (PMC5875991; doi:10.3390/metabo8010001)
Supplement: Supplementary file 1 [file metabolites-08-00001-s001.zip › metabolites-08-00001-supple/FigS1_FigureAbundanceHistograms.pdf]

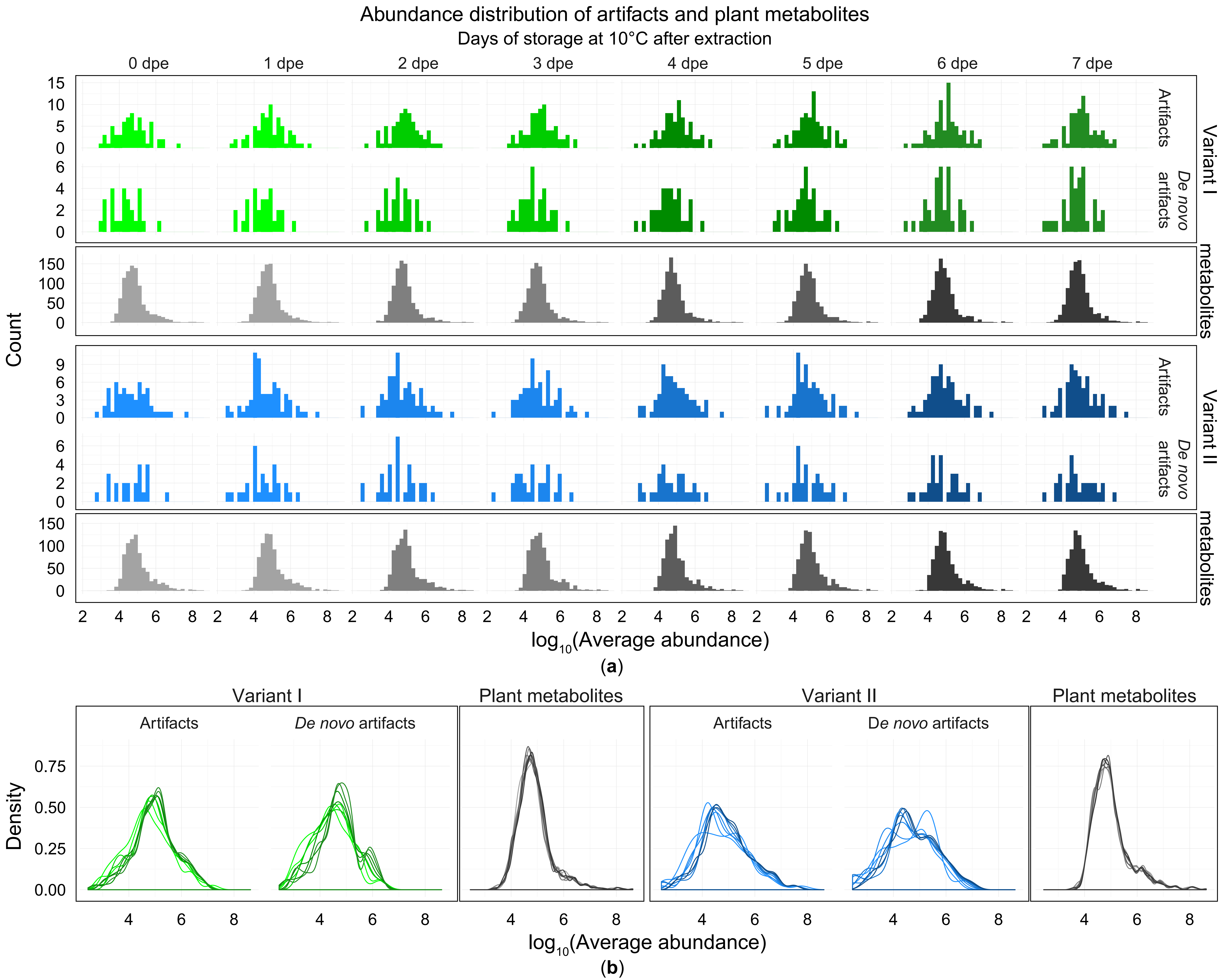

Figure S1. (a) Illustration of the mean abundances of all artifacts, *de novo* formed artifacts (green color gradient for variant I, blue color gradient for variant II) and all plant metabolites (grey color gradient; from additional experiment) after extraction with acidified aqueous methanol (top three rows) and pure aqueous methanol (three rows below) for each time point within the first week of storage at 10°C. (b) Density plots of mean abundances for 0 dpe to 7 dpe for all artifacts, *de novo* artifacts and all plant metabolites for variant I and II.
